# Perceived Acceptability of SPACE for COPD© as a Maintenance Option Following Pulmonary Rehabilitation Discharge: A Qualitative Interview Study with Patients and Facilitators Using Framework Analysis

**DOI:** 10.2147/COPD.S538733

**Published:** 2026-04-23

**Authors:** Amy C Barradell, Khaled Alqahtani, Annabel Hong, Jessica Lapworth, Kathryn Greenall, Israa Al-Naabi, Ala Szczepura, William D C Man, Claire M Nolan, Gillian Doe, Nikki Gardiner, Charlotte Gerlis, Claire Bourne, Sally J Singh, Linzy Houchen-Wolloff

**Affiliations:** 1 NIHR Respiratory Biomedical Research Centre, University Hospitals of Leicester NHS Trust, Leicester, UK; 2 School of Psychology and Vision Sciences, University of Leicester, Leicester, UK; 3 Department of Respiratory Sciences, University of Leicester, Leicester, UK; 4 School of Physiotherapy, University of Leicester, Leicester, UK; 5 Centre for Healthcare and Communities, Coventry University, Coventry, UK; 6 Harefield Respiratory Research Group, Guy’s & St.Thomas’ NHS Foundation Trust, London, UK; 7 King’s Centre for Lung Health, King’s College London, London, UK; 8 National Heart and Lung Institute, Imperial College, London, UK; 9 College of Health, Medicine and Life Sciences, Brunel University of London, London, UK; 10 Centre for Health and Clinical Research, University of the West of England, Bristol, UK

**Keywords:** COPD, pulmonary rehabilitation, self-management, motivational interviewing, qualitative research

## Abstract

**Introduction:**

Following completion of Pulmonary Rehabilitation (PR), the biopsychosocial benefits often decline. PR maintenance is recommended to extend the benefits; however, detail lacks on what this should entail. SPACE for COPD© is a light touch, evidenced-based self-management intervention which we tested as a maintenance strategy during the COVID-19 pandemic. We explored the acceptability of SPACE for COPD© as a maintenance option following PR for both patients and facilitators using qualitative research methods.

**Methods:**

We conducted semi-structured interviews and focus groups with patients and intervention facilitators involved in the SPACE for COPD© maintenance study. These were audio recorded, transcribed verbatim and analysed using Framework Analysis.

**Results:**

Seventeen patients were interviewed; 13 (76.5%) of which were programme completers: five (29.4%) received a group-based intervention, four (23.5%) received one-to-one, and eight (47.1%) received hybrid. Two focus groups were conducted with eight facilitators; six (75%) were physiotherapists, one (12.5%) was a nurse, and one (12.5%) was a health psychologist. Analysis generated six themes: (1) the changing structure of maintenance SPACE for COPD©; (2) the integral role of the facilitator; (3) engagement with the manual required time and commitment from patients; (4) staying active on maintenance SPACE for COPD© was facilitated by goal setting and exercise; (5) biopsychosocial outcomes of maintenance SPACE for COPD©; and (6) the future of maintenance SPACE for COPD©.

**Conclusion:**

Maintenance SPACE for COPD© was acceptable and could be implemented into the PR healthcare pathway during the COVID-19 pandemic. It helped patients to adopt and integrate new exercise habits if they could adapt and personalise these to their home lives. Adaptions made during the COVID-19 pandemic created a menu of options. Building upon this personalisation is required to ensure the intervention is accessible and patient-centred.

## Introduction

Pulmonary rehabilitation (PR) is offered to individuals living with Chronic Obstructive Pulmonary Disease (COPD) to optimise their respiratory health and wellbeing.^1^ Traditionally, the programme includes at least six weeks of supervised and progressive exercise and self-management education. When individuals complete the programme, they receive an action plan to guide them in maintaining their health and wellbeing. Efficacy research shows that individuals who complete PR experience a significant improvement in their exercise capacity, dyspnoea, fatigue, emotional wellbeing and sense of control over their COPD.^1^ However, without the ongoing support of the programme, some individuals describe a sense of “abandonment”^2^ and the aforementioned benefits can quickly diminish.^3^

The American Thoracic Society (ATS) and the British Thoracic Society (BTS) suggest supervised maintenance PR may be beneficial to sustain the benefits of PR. However, owing to a lack of evidence for maintenance programmes, no recommendations describe exactly what this should entail.^4^,^5^ The ATS and BTS have requested further research to determine the required frequency, duration, content, and amount of supervision for both supervised and unsupervised maintenance options to better inform the prescription of maintenance PR.

Our research group has developed and extensively tested the SPACE for COPD©, a “Self-management Programme of Activity, Coping and Education,” for individuals living with COPD.^6^ The intervention consists of a four-stage self-management manual, SMART goal setting sheets, and four group-based sessions delivered three months apart and facilitated by respiratory healthcare professionals trained in motivational interviewing.^6^ Across differing PR populations and contexts, it has shown to improve individuals’ symptoms of COPD, exercise tolerance, and quality of life.^6–8^ Post-intervention, qualitative interviews have also shown that individuals continued to use the manual after the study had finished.^6^ This demonstrates the promise of SPACE for COPD© as a maintenance option. However, owing to a lack of research, its suitability as a maintenance option is largely unknown.

The maintenance SPACE for COPD© study was therefore conducted to evaluate whether SPACE for COPD© was an effective maintenance option for individuals living with COPD following completion of PR. The intervention is detailed in full in our published protocol.^9^ The results showed it was cost-effective as it significantly reduced General Practice costs in the intervention group. However, there were no significant differences between the groups for exercise capacity, quality of life, psychological wellbeing, patient activation or physical activity.^10^ Here we report the qualitative evaluation. We sought to collect qualitative appraisals from both the people who participated in the study and the healthcare professionals who facilitated the intervention’s delivery to inform acceptability and implementation of the intervention. Our objectives were to explore the barriers and facilitators to patients’ maintenance of wellbeing and appraisals of their sense of abandonment.

In writing this article, the consolidated criteria for reporting qualitative research (COREQ) guideline has been used to ensure transparency of reporting and reproducibility of the study^11^ (see Supplementary Material, S1).

## Methods

### Study Design

This was a dual-centre, randomised controlled trial with nested qualitative interview and focus group study^9^ using an interpretativist approach.^12^ Qualitative methodology was adopted to support rich data collection specifically related to the participants’ experiences of the study^13^ We utilised individual interviews for patients and focus groups for facilitators who delivered maintenance SPACE for COPD©.

### Research Team and Reflexivity

The qualitative research team was led by a female Health Psychologist experienced in qualitative healthcare research and situated within a respiratory research team (AB) and a male PhD student with qualitative research experience in respiratory sciences (KA). The qualitative analysis team consisted of three female trainee physiotherapists who were new to qualitative methods (JL, KG, and IA). None of the research team had any involvement in the intervention delivery.

### Participant Selection

Patients were eligible if they had been recruited to the maintenance SPACE for COPD© study and had opted in to the qualitative interview on their informed consent form. Prior to each interview, patients completed a further consent form for this nested part of the study. We utilised the maximum variation sampling^14^ to consider research site (ie Leicester or Harefield), sessions received (ie group sessions, one-to-one sessions, hybrid sessions), and completion status (ie completers and non-completers). Completers were those who attended all four intervention sessions. Non-completers attended between zero and three sessions. We included both completers and non-completers to capture a wide range of perspectives.

Intervention facilitators were eligible to participate in the focus group if they had experience of the maintenance SPACE for COPD© training, as detailed in the protocol^9^ and delivered the intervention sessions.

### Setting

Patients were included from both research sites. Qualitative interviews and focus groups were conducted by Leicester research team.

### Data Collection

For the interviews, we developed an interview schedule that could be used flexibly to respond to unanticipated issues should they arise (see Supplementary Material, S2). This schedule was slightly revised following initial interviews to capture appraisals of the varying intervention delivery methods (eg face to face, online, telephone, group, 1–1) which occurred as a result of the COVID-19 pandemic questions focused on our objective which was to explore the implementation of SPACE for COPD© as a maintenance strategy Interviews sought to capture any barriers and facilitators to patients’ maintenance of wellbeing and appraisals of their sense of abandonment.

Similarly for the focus groups, we developed an interview schedule that could be flexibly used to respond to unanticipated issues (see Supplementary Material, S3). This enabled an iterative approach, allowing amendments in response to the COVID-19 pandemic and changes in intervention delivery methods. Questions sought to explore the associated burden and benefits of implementing the intervention into the PR healthcare pathway.

Semi-structured interviews were conducted by AB with patients in the intervention group 12 months post-enrolment onto the study or before if a participant decided to discontinue their intervention. It was intended these would be face to face at hospital or the patients’ home. However, the majority were conducted during the COVID-19 pandemic, and so many were telephone interviews. One participant described themselves as “hard of hearing” and could not complete the interview via telephone and so opted to provide written answers to the interview questions instead.

Focus groups were conducted by KA with intervention facilitators after completing their training and late on in intervention delivery. AH also attended to take field notes. As intended, these were conducted using videoconferencing software.

We proposed a sample size of 10–20 patients and 5–10 intervention facilitators (in two focus groups) or until it was assessed that data saturation^15^ had been met. Interviews and focus groups were audio recorded and transcribed verbatim. Descriptive and reflexive field notes were taken to provide additional context and support interpretation and analysis.

### Data Analysis

We used the framework analysis approach for data analysis.^16^ This approach allowed us to have pre-set objectives (detailed above) whilst ensuring appraisals reflect participant experiences. We followed the five steps of framework analysis: familiarisation with the data, identification of a thematic framework, indexing the transcripts, charting the data to the thematic framework, and interpretation of the framework. We used Microsoft Excel to index the data within the thematic framework. All steps were conducted by KA, JL, KG, and IA and overseen by AB. Final interpretation of the framework was led by AB with support from all authors.

## Results

A total of 35 patients agreed to hear more about this qualitative interview study. Of these, 23 (65.7%) patients were selected for interview using the maximum variation sampling method and of those 17 (73.9%) consented. Interviews were conducted face to face (n=3), via telephone (n=12), via videoconferencing (n=1), or via written response (n=1). They lasted between 27 and 96 minutes. Table 1 details the demographics of the patients.

**Table 1:** Demographics of Interview Patients

Study ID	Status	Delivery	Gender	Age (yrs)	Ethnicity	Employment Status	Level Reached in Education	Smoking Status
H04	Completer	Hybrid	Female	91	White British	Retired	University Bachelors	Never Smoked
H06	Completer	Hybrid	Female	71	White British	Part-time	Secondary School	Ex-smoker
H12	Completer	Hybrid	Female	82	White British	Retired	Secondary School	Ex-smoker
H14	Completer	Hybrid	Male	58	White British	Full time	Secondary School	Ex-smoker
H15	Completer	Hybrid	Female	64	White British	Full time	Secondary School	Ex-smoker
H20	Completer	Hybrid	Male	78	White British	Retired	Secondary School	Ex-smoker
H35	Non-Completer	One to one	Female	76	White British	Self-employed	Secondary School	Ex-smoker
H46	Completer	Group	Male	75	White British	Retired	Secondary School	Ex-smoker
H49	Completer	Group	Male	83	White British	Retired	Secondary School	Ex-smoker
H48	Completer	Group	Male	83	White British	Retired	Secondary School	Ex-smoker
G02	Completer	Hybrid	Male	71	White British	Retired	University Bachelors	Ex-smoker
G03	Completer	Hybrid	Male	63	Mixed White and Black Caribbean	Long term sickness	Secondary School	Current Smoker
G11	Non-Completer	One to one	Male	86	White British	Retired	Secondary School	Ex-smoker
G12	Non-Completer	One to one	Male	81	White British	Retired	Secondary School	Ex-smoker
G19	Completer	One to one	Male	75	White British	Retired	Secondary School	Ex-smoker
G29	Non-Completer	Group	Female	63	White British	Unemployed	Secondary School	Ex-smoker
G37	Completer	Group	Female	67	White British	Retired	Secondary School	Ex-smoker

Two videoconference focus groups were conducted with intervention facilitators. The first was conducted on the 16^th^ August 2022 (n=3) and the second on the 31^st^ August 2022 (n=5). Table 2 details the demographics of the facilitators.

**Table 2:** Demographics of Focus Group Facilitators

Study ID	Professional Background	Type of Sessions Delivered
HCP1	Physiotherapist	Hybrid
HCP2	Physiotherapist	Did not deliver maintenance SPACE for COPD©, attended training only
HCP5	Physiotherapist	Hybrid
HCP6	Physiotherapist	Hybrid
HCP7	Health Psychologist	Hybrid
HCP8	Physiotherapist	Group
HCP9	Nurse	Group
HCP10	Physiotherapist	Group

The analysis generated six themes and thirteen sub-themes: 1) the changing structure of maintenance SPACE for COPD©; 1a) Experiences of an interactive group approach vs 1–1 tailored approach; individual preference is key; 1b) A minority had little understanding and recall of maintenance SPACE for COPD©; 1c) Transport and access to the changing delivery methods was highly varied; 2) the integral role of the facilitator; 2a) Credibility and skill of facilitator and resources used provided holistic and effective healthcare; 2b) The therapeutic relationship was important for positive behaviour change; 3) engagement with the manual required time and commitment from patients; 3a) Layout of the maintenance SPACE for COPD© manual was acceptable to those with good literacy skills; 3b) How the maintenance SPACE for COPD© manual was used (inside and outside of sessions); 4) staying active on maintenance SPACE for COPD© was facilitated by goal setting and exercise; 4a) Having the right resources and environment was important to support goal setting and exercise; 4b) Understanding the importance of exercise motivated goal setting and exercise; 4c) The importance of meaningful and realistic goals; 5) biopsychosocial outcomes of maintenance SPACE for COPD©; 6) the future of maintenance SPACE for COPD©; 6a) A menu of maintenance options is needed to meet differing needs; 6b) The maintenance SPACE for COPD© manual can be more appropriate for those with COPD; and 6c) Access to healthcare professional and social support is an essential component of any maintenance SPACE for COPD©.

The themes, along with their sub-themes, are presented below with illustrative quotes. Quotes with a “H” or “G” attached indicate a quote from a participant from either the Harefield or Glenfield site, and a “HCP” indicates a quote from an intervention facilitator.

**Figure 1:**
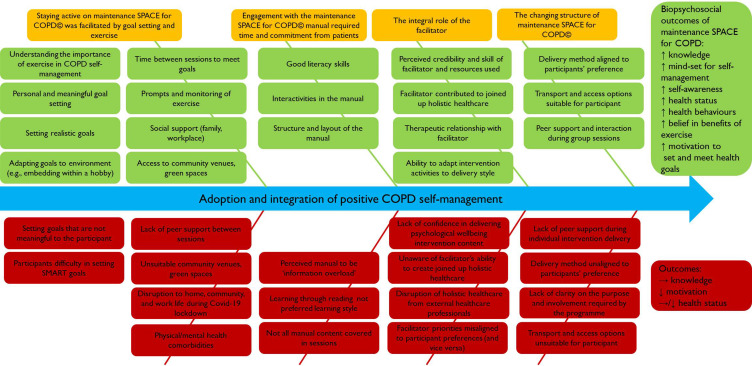
Barriers and facilitators to the adoption and integration of positive COPD self-management represented along a continuum towards the biopsychosocial outcomes (yellow items represent the themes involved in the adoption and integration of positive COPD self-management, green items denote the facilitators and red items denote the barriers).

Figure 1 highlights the barriers and facilitators to the adoption and integration of positive COPD self-management from the perspective of patients and maintenance SPACE for COPD© facilitators. The theme “The future of maintenance SPACE for COPD©” is not included in Figure but is detailed in the results section below.

### The Changing Structure of Maintenance SPACE for COPD©

Differing delivery methods of maintenance SPACE for COPD© were adopted out of necessity in response to the COVID-19 pandemic. Some patients had group sessions either face-to-face or via teleconferencing facilities, whereas others had 1–1 sessions either face-to-face or via telephone. Facilitators reflected that these adaptations were important to “offer people some kind of discussion with a healthcare professional during a really scary time” (HCP1; hybrid sessions), but felt they had to adopt creative delivery styles to adapt the activities to the differing delivery methods.

This theme encompasses how patients felt about the intervention that they received and what worked or did not work well for them.

#### Experiences of an Interactive Group Approach vs 1-1 Tailored Approach; Individual Preference is Key

For those who received the group sessions face to face or via teleconferencing, patients and facilitators enjoyed the physical interaction with group members as it created a sociable atmosphere in which patients were comfortable to connect, offer advice, and share their experiences of living with COPD with the group. This reassured them they were not alone with their COPD and provided practical and empathetic discussion of challenges and how to overcome these in self-management.

Patients were able to talk openly about their own experiences, for example, going into hospital and ignoring their symptoms of an exacerbation so that was quite powerful for other patients. (HCP8; group sessions)

Having four men in the room discussing themselves and their attitudes to things was quite illuminating. (H48; group sessions)

When someone else has got the same problem as you… You can get it off your chest and they in turn will relate to you… you feel more normal. (H12; hybrid sessions)

Facilitators further described the atmosphere as one of equality, in which patients and facilitators created a cohesive group which took away the hierarchy of roles. This was established using “ground rules” (HCP1; hybrid sessions) which aligned to the ethos of motivational interviewing in the first session.

I feel a lot more like it was just a group of all of us together delivering it rather than that kind of separation of healthcare professional and patient. (HCP6; hybrid sessions)

Patients and facilitators described the structure of the sessions as consistent but flexible enough to react to patients’ contributions and interactions. They also provided the opportunity to ask the facilitators questions and a chance to provide feedback to them.

…we didn’t actively move any content around but sometimes things come up… [For example] if somebody comes in who appears to be having a raging flare up, then you might need to consider talking to him about recognising their symptoms on session one. (HCP8; group sessions)

Every so often something was thrown in…like a general discussion than a specific programme. (H20; hybrid sessions)

On the other hand, facilitators and some patients disclosed concerns about the sharing of very personal thoughts and feelings with the group during the discussions about anxiety and depression. Facilitators wondered whether patients would be more comfortable having this discussion on a one-to-one basis. Additionally, some patients found that a contrast of characters in the group or patients discussing irrelevant topics was off-putting and difficult to relate to. Facilitators found it “tricky” (HCP8; group sessions) at times to move the discussion along.

Some people see [anxiety or depression] as a weakness or a stigma, so they don’t want to admit where they might have issues especially in a group because they think they’ll get labelled. (HCP5; hybrid sessions)

The old fella would talk nothing but about his life… You’re not there for that. (H46; group sessions)

For those who received the intervention via telephone, patients and facilitators described the value in the personalisation and tailoring of their interaction. This enabled them to ask specific questions pertinent to them and their needs.

I think for the one to one sessions, because you were focusing just on one person, then it was probably a lot easier… for me it was quite an easy flow. (H15; hybrid sessions)

…I did like over the phone… it allows more time for focus on that person. (HCP7; hybrid sessions)

However, patients and facilitators found that the telephone sessions could become repetitive and at times restricted the flow of the conversation.

The con by telephone is obviously there’s no interaction because there’s only the two of you… it tends to be really short, perhaps 20 minutes, half an hour. (HCP1; hybrid sessions)

You can only say so much over the phone… There’s something missing. (H20; hybrid sessions)

Facilitators found this method of intervention delivery removed the element of peer support which changed the dynamic of the interaction back to one involving hierarchy.

[The 1-1 sessions] puts some patients in what we call in motivational interviewing, that pleasing the teacher situation… so they’re doing things because you’re ringing them. (HCP7; hybrid sessions)

#### A Minority Had Little Understanding and Recall of Maintenance SPACE for COPD©

Some patients felt that they did not fully understand, could not recall, or could not differentiate the maintenance SPACE for COPD© or manual from PR. They felt their understanding was significantly impaired by the length of time between sessions making previous content difficult to recall, if they missed the first session, or they were experiencing underlying stress which took their attention away from the intervention.

Every four months, it takes some doing to remember what you done on that session… (H46; group sessions)

I do remember a telephone call… I’m sure it’s probably to do with the study. I don’t know for sure because my head’s all over the place. (H35; one-to-one sessions)

#### Transport and Access to the Changing Delivery Methods Was Highly Varied

In response to the COVID-19 pandemic, the facilitators at one site described numerous venue changes which created differing logistics and environments for both them and patients to adapt to. Due to this, some patients found that face-to-face sessions were easy to access with adequate facilities and parking at the sites while others had great difficulty parking at, and accessing the sites which caused anxiety for them. Other patients relied on public transport which was insufficient and made it difficult to attend on time.

Parking is horrendous…I was rushing, and I was a bit anxious… I thought I’m going to be late. (H15; hybrid sessions)

…the last venue we were back on site but a different venue to the original one and It actually worked out really well for patients because they could park right outside. (HCP1; hybrid sessions)

Once at the group sessions, some patients felt the need to wear face masks made it difficult for them to engage in the session as it reduced their ability to observe and engage in non-verbal communication. It also negatively impacted their COPD symptoms

You can’t actually see the face that you want to see and see their expressions. (H46; group sessions)

For those who received the intervention via telephone calls and teleconferencing, both patients and facilitators described its ease as it could be done flexibly from home which was pertinent to those who were advised to shield during the COVID-19 pandemic and those with scheduled commitments to work around.

[The 1-1 calls were] more convenient being at home because you don’t have to worry about getting buses. (G02; hybrid sessions)

[If] for pandemic reasons they’re not allowed to come in then it still offers them the choice to still have the session but done remotely. (HCP5; hybrid sessions)

Patients felt safe because they were not around others during the COVID-19 pandemic, and for those who could and wanted to access the internet, they found the technology easy to use.

The main reason I wanted it on the phone was COVID. I didn’t want to go into a room where people have got COVID. I’ve already had it, I don’t want it again. (G19; one-to-one sessions)

Some of the groups didn’t have access to the internet or didn’t want to do the session via video conference. (HCP1; hybrid sessions)

For one person who received the intervention via teleconference, they described their ease in joining the session but the difficulty facilitators faced to use the required technology.

I’m quite used to skyping… [but the facilitator] had trouble… getting it to work. (H12; hybrid sessions)

### The Integral Role of the Facilitator

This theme articulates how the facilitators’ role influenced the way patients engaged with maintenance SPACE for COPD© and their overall COPD management.

#### Credibility and Skill of Facilitator and Resources Used Provided Holistic and Effective Healthcare

Patients described their gratefulness to have facilitators with expert knowledge and experience in COPD management. This made them appear approachable both during and outside of sessions to discuss topics relevant and valued by the patients.

[Facilitator] was at the pulmonary course I was on anyway, so I knew her anyway. (H46; group sessions)

Likewise, facilitators reflected on their professional backgrounds which they felt brought a variety of specialisms to the intervention.

I really liked delivering the sessions. I think I was coming to them with a slightly different mindset, maybe because I had more experience in managing the MSK based long term conditions. (HCP6; hybrid sessions)

Facilitators expressed their growth in supporting the self-management of patients through the intervention’s training, the facilitator manual, and the use of motivational interviewing.

I’ve been to the training, motivational interviewing, but just to practically do it in the group session like this is something new… I think the feedback from [facilitator name] has been really useful. (HCP10; group sessions)

[The facilitator manual] was really good to provide structure to your session, but also because we had new staff joined like [facilitator name], for example, it made my job in terms of teaching him really easy. (HCP1; hybrid sessions)

Furthermore, facilitators felt that working with other facilitators and patients made them more-rounded healthcare professionals.

I’ve delivered sessions with most of the people on this call now and seeing how people bring their different sort of skills and experience to the sessions has been really interesting… I’ve learned a lot. (HCP5; hybrid sessions)

However, the changes incurred in response to the COVID-19 pandemic made facilitators anxious regarding their ability to fully deliver the intervention as it was intended. Additionally, without a background or experience in discussing psychological wellbeing with COPD patients, some facilitators found themselves questioning their appropriateness to deliver this. This was not echoed by patients.

[It was challenging to] get them to think about acceptance of a different situation when we hadn’t trained for that. (HCP7; hybrid sessions)

The resources available to facilitators, for example, their ability to facilitate ongoing referrals to smoking cessation services and the maintenance SPACE for COPD© manual, bolstered patients’ appraisal of their credibility.

We had the manual with us at all times so we could sort of put a little bookmark in for them to say you might want to read a bit more on pages xx. (HCP5; hybrid sessions)

Patients described this as creating a joined-up service which focussed upon holistic and effective healthcare. It allowed them multiple resources to approach when they required support for their COPD symptoms. It also reinforced the value of positive self-management which was important to help patients prioritise their health.

I feel if I hadn’t taken the help [from pulmonary rehabilitation, SPACE [for COPD©] maintenance, and bereavement counselling] I’d still be down there…and it’s made me realise my health’s important. (G37; group sessions)

On the other hand, some patients were unaware that they could contact the facilitators or felt they did not want to bother facilitators between sessions which lowered their perception of the facilitators’ support in their self-management. Additionally, some facilitators did not openly offer this support.

I haven’t wanted to bother people really, I mean especially in the National Health Service with all that’s going on at this moment in time. (H12; hybrid sessions)

For clarification… you would advise patients to call if they have any queries, right? No, there was no specific advice to do that. (HCP1; hybrid sessions)

Without optimised healthcare, facilitated by a joined-up service, patients found inconsistencies arose and this caused distress as they no longer felt they had complete access to holistic and effective care.

I had to change [primary care] doctor… That was very upsetting because it was great having a fantastic doctor. (H35; one-to-one sessions)

#### The Therapeutic Relationship Was Important for Positive Behaviour Change

Having a therapeutic relationship with the facilitators made patients feel at ease and cared for, helped to motivate them to achieve their goals and provided self-recognition and positive reinforcement when goals were achieved.

I felt like somebody was really looking out for me. (H06; hybrid sessions)

She used to tell you what she’d done and I’d say right I’m looking to smash your numbers then. (G03; hybrid sessions)

I told [facilitator] that I’d done it and she was over the moon I’d done it so quick. (H46; group sessions)

Likewise, seeing the patients progress and supporting this progression was enjoyable for the facilitators.

I like helping people and getting them to see what they’ve done and moving them forward. (HCP7; hybrid sessions)

However, the therapeutic relationship was challenged when facilitators’ actions did not align with participant preferences. For example, facilitators wanted to share individuals’ goal achievements during the group sessions but not all patients felt comfortable with this.

She said, can I say it to the class? I said no, don’t tell anybody. (H46; group sessions)

### Engagement with the Maintenance SPACE for COPD© Manual Required Time and Commitment from Patients

Patients had varying engagement levels with the manual, which they described as being mediated by the time and commitment they were prepared to put into it.

#### Layout of the Maintenance SPACE for COPD© Manual Was Acceptable to Those with Good Literacy Skills

The layout of the manual was perceived by some patients as being very informative with interactive components which supported continued engagement. It was also described as “well laid out” (H15; hybrid sessions) and “easy to read” especially because of the “big writing” (H14; hybrid sessions).

It’s got testimonials from other people and how they felt. (H06; hybrid sessions)

There were pictures in it and you had to try these different pictures, then mark against the ones you could do. (G11; one-to-one sessions)

However, other patients found it was too large, heavy, an overload of information, or provided in the wrong format. Some patients did not feel it was worth keeping the manual once the intervention was over.

I don’t do an awful lot of reading… I prefer to be more practical. (H12; hybrid sessions)

#### How the Maintenance SPACE for COPD© Manual Was Used (Inside and Outside of Sessions)

The manual was frequently referred to as a reference which patients could “look back on” (H20; hybrid sessions) as they needed. Some patients integrated it into their daily routine (eg via bedtime reading), others used it to guide them when doing breathing exercises and to record their exercise progression which was useful for patients and facilitators to monitor goal progression. Additionally, some patients found it useful to write down questions for the facilitators to ask them at the next session. However, some patients described their exercise progression records as not always reviewed by facilitators which reduced their desire to engage with the manual.

I filled in what I was doing each day… but nobody took any notice of that, so I thought there’s no point me carrying on with it. (H46; group sessions)

Additionally, some patients felt that not enough of the manual content was covered during the sessions, and they wished for more time with the facilitator to go through it.

When you see [facilitator] about three times a year you’re just going through a couple of bits. (G37; group sessions)

### Staying Active on Maintenance SPACE for COPD© Was Facilitated by Goal Setting and Exercise

This theme encompasses the factors necessary for the adoption and maintenance of goal setting activities and exercise.

#### Having the Right Resources and Environment Was Important to Support Goal Setting and Exercise

Patients and facilitators described the importance of having time between the sessions for patients to work on their goals, so they could make progress in time for the next session. However, some patients felt they were too far apart and the lack of peer support during this time, particularly for those receiving the 1–1 sessions, was demotivating.

Without talking to others about exercise the idea will slip away again. (G03; hybrid sessions)

Facilitators felt that the cohesion established during the group sessions was motivational and supported goal attainment but that this was lost in the one to one sessions.

[In the 1-1 sessions] they’re doing things because you’re ringing them. Whereas in a group setting that group cohesion and doing things together and having us more as like facilitators builds that kind of confidence to do things. (HCP7; hybrid sessions)

Patients found the manual helpful to monitor their progression between sessions, with some developing their own complementary prompts to remind them to exercise.

I do have my own programme… I can have it on the table when I’m having breakfast to remind me. (H04; hybrid sessions)

Some patients felt they had suitable equipment and environment to exercise at home whereas others did not. This included having the support of family members to help them detect and monitor their COPD symptoms. Some patients described their workplace managers as supportive as they facilitated more time for exercise or self-management, as needed. Likewise, some patients felt access to community venues, green spaces, or virtual platforms particularly during the COVID-19 lockdowns provided the right resources and environment to exercise.

Joe Wicks, that’s it, that’s the one I’ve been doing… They’re only 15 minutes twice a day. (H12; hybrid sessions)

One considerable barrier to both access of community venues and desire to set, adhere to, and record goal progression was the COVID-19 pandemic. During lockdown, patients described significant changes to their home and work lives which disrupted their activity regimes, motivation levels, well-being and prompted the re-adoption of negative health behaviours (eg smoking).

I did start losing [weight] and then of course everything went bottoms up. (H20; hybrid sessions)

I couldn’t exercise because I was shielding. (H15; hybrid sessions)

Facilitators also recognised that despite previous achievements, the COVID-19 pandemic “… massively set them back” (HCP7; hybrid sessions) and attributed this to patients’ deteriorating psychological wellbeing during this time.

Other barriers included either too hot or too cold weather, paying for parking at community venues/green spaces, lack of resting spots in green spaces, feeling out of place in community gym venues, inability to stabilise a COPD symptom, lack of family or social support and additional physical or mental health comorbidities which limited patients desire and ability to engage in exercise and self-management. At times, this was due to medication interactions.

I’ve been taken a steroid for my MS and I think that’s the thing that’s affected the chest, so it’s made it difficult for me to do anything for the last few days. (H46; groups sessions)

Facilitators from one site were concerned that the exercises and action plans in the maintenance SPACE for COPD© manual differed from their site’s procedures and could create confusion for patients; however, this concern was not echoed by patients.

#### Understanding the Importance of Exercise Motivated Goal Setting and Exercise

Patients reflected on their awareness of the benefits of exercise via what they had learned in the sessions, experientially through a long-standing physical activity hobby, a relative’s experience of COPD, PR, or their goal achievements.

[I’ve learned that] carrying on exercising and stuff like that is going to be of benefit, which is why I’ve carried on doing it. (G02; hybrid sessions)

When I finished pulmonary rehabilitation I found I was very motivated to keep everything up. (H15; hybrid sessions)

Patients described this awareness leading to beliefs such as if one maintains their physical activity this will reduce the number of hospital admissions and therefore NHS expenditure, or that exercise is an important part of COPD self-management. If patients related these beliefs to their own life and the personalised meaning it would bring, this increased their motivation to engage in goal setting to further strengthen their exercise capacity.

…I want to be here for my daughter and I want to be here for my grand-son… so I’m more bothered about my health. And one of the reasons for coming on this programme is because I wanted to help myself. (G37; group sessions)

However, some patients did not reach these beliefs and instead felt other areas of their life required greater priority. Or that the COVID-19 pandemic reduced the meaningfulness of maintaining their exercise capacity or goal setting activity.

I think there were exercises in the book that she said to do. I was lazy and didn’t do them… I was at home doing nothing. (H14; hybrid sessions)

#### The Importance of Meaningful and Realistic Goals

Patients described the importance of crafting personal goals that would be meaningful to their life circumstances. This was achieved by identifying areas of their life important to them and creating goals which aligned. Some patients chose to align goals to their current lifestyle, for example, by embedding their goal within a physical activity or hobby.

I’ll book holidays ahead… And to look forward to something you’ve got to work for it and if that means getting my health back a bit more then fine, I’ll do it. (G19; one-to-one sessions)

[I] always go for a walk every morning with the dog for about 40 minutes, rain or snow doesn’t worry me, she’s got to go out. (H20; hybrid sessions)

Other patients described making changes to their lifestyle to support their adoption of a goal, for example, doing exercises with a family member, flexibly adapting exercises to their ability or the environment available to them.

I’ve just changed my ways, not eating late and eating early in the morning when I get up instead of picking all day… (G29; group sessions)

The achievement of initial goals led to the setting of further goals by patients and facilitators. This helped patients to make consistent changes that continued to be important to them.

You just keep doing it all the time, the more goals you make as you go along, if you complete one make a new one. (G19; one-to-one sessions)

However, when goals did not align with a patients’ life circumstances these appeared more difficult to recall and less appealing to commit to.

Lockdown has interrupted my memory. (H12; hybrid sessions)

The one topic that no one ever had anything to say about was starting up a new hobby. (HCP1; hybrid sessions)

Particularly during the COVID-19 pandemic, facilitators felt that patients struggled to set meaningful goals because of all the limitations in place.

… it was harder for [the patients] to think about things they could do. So they have to do a lot more problem solving. (HCP7; hybrid sessions)

Additionally, patients reflected on the importance of creating achievable goals. Whilst there was temptation to create unrealistic goals to improve their health more quickly, when these were not met they caused disappointment.

I just didn’t achieve any… she actually did say it was quite ambitious. (H15; hybrid sessions)

Facilitators recalled some patients having difficulty in understanding and therefore creating goals which aligned to the SMART criteria (ie specific, measurable, achievable, realistic, and timely). Not only did patients have difficulty creating realistic goals, they also struggled to create specific goals.

To set SMART goals, the smart aspect of it was really difficult… People have set goals because they’ve been to rehab, but being so specific about it, I think it was challenging for some people. (HCP1; hybrid sessions)

### Biopsychosocial Outcomes of Maintenance SPACE for COPD©

This theme captures patients’ perceived physical, psychological and social outcomes from taking part in the maintenance SPACE for COPD©.

Some patients described improved knowledge and mind-set for their COPD management which led to increased self-awareness of their own body and overall health status. These changes led to taking steps to better self-manage (eg by stopping smoking, continuing to set goals, adoption of techniques learnt during the intervention into their day-to-day management). For example, one participant described how they would now manage a panic attack. Another described adopting “blow as you go” as they knew it would be a useful tool to help them climb the stairs.

I couldn’t go up the stairs without stopping… [facilitator] said ‘take a nice deep breath… blow through pursed lips…’ you suddenly realise how important… and how much it helps. (H06; hybrid sessions)

Patients described both themselves and family member’s recognising physical and mental health improvements from the intervention which further increased their beliefs in the benefits of exercise and their desire to set and meet their goals.

I love to walk because it takes your mind off things. (G37; group sessions)

Through exercise I’ve lost a lot of weight… I feel a lot fitter. (G02; hybrid sessions)

However, some patients felt that they did not gain any new knowledge from taking part in the intervention or felt that most of the information was taught during PR so there was little more to learn in the maintenance intervention. Some patients also had varying success continuing with their goals following the intervention as their motivation diminished without the supervision and peer support offered in the group sessions.

I’m still trying but I don’t seem to be losing as much as quickly now… Sometimes we don’t talk about things often enough and I suppose when we talk to other people it’s a great motivator to do something about it. (H48; group sessions)

### The Future of Maintenance SPACE for COPD©

This theme captures appraisals of the future of maintenance SPACE for COPD© and what it should entail.

#### A Menu of Maintenance Options is Needed to Meet Differing Needs

Patients and facilitators both recognised the value in providing a menu of maintenance options to support uptake and engagement, similar to those delivered in response to the COVID-19 pandemic. Patients described differing preferences for the level of facilitator contact they required for the intervention. Some patients preferred the manual without supervision, whereas others preferred a tailored 1–1 supervised approach, or a supervised group environment. Additionally, patients expressed varying preferences for how long and how it should be delivered, with some preferring face to face approach and others preferring telephone or online approaches.

I would have carried on coming up to the hospital [for group sessions] given the chance. (G02; hybrid sessions)

I think the one-to-one is quite good as well. But then again some people would prefer to just have the book… (G29; group sessions)

Facilitators speculated on the size of the group sessions, whilst acknowledging the small group sizes were less cost-effective, they were concerned that larger group sizes (as originally intended) would reduce some patients desire or confidence to contribute to the discussions.

I could sort of imagine that in a bigger group in the maintenance programme that people [would be] either not interested or really quiet. (HCP10; group sessions)

Some patients thought there would be value in adapting the intervention to a live telehealth option, or an online or smartphone application, but recognised this may not be suitable for all digital literacy levels.

I know a lot of younger ones have the Kindle books. Well you could do it in that sort of form. (G37; group sessions)

Other patients thought the current intervention was suitable and were interested in repeating it or having access to group, face-to-face sessions now that the COVID-19 lockdowns had been lifted. Others patients suggested minor adaptations such as the addition of biofeedback devices to monitor exercise progress or to incorporate supervised exercise into the intervention to boost motivation for exercise outside of the sessions.

The thing that I benefitted most from is the physical activities on the rehabilitation course prior to the research programme. So if there was a little bit more emphasis on that, rather than talking about it and then hoping that I was going to do it when I left the room… It might make a difference. (H48; group sessions)

#### The Maintenance SPACE for COPD© Manual Can Be More Appropriate for Those with COPD

As described above, the manual was described as not suitable for all and so patients felt there would be value in simplifying it through the addition of bold statements highlighting the key self-management messages. Additionally, patients felt adding more information about the stages of COPD severity and case studies to illustrate how others have self-managed may make it more relatable and useful.

There could be a page where from experience, say from other people… ‘You will feel better because we’ve already proved it because Mr So and So or Mrs So and So did this, that and the other and it improved their situation’. (H35; one to one sessions)

#### Access to Healthcare Professional and Social Support is an Essential Component of Any Maintenance SPACE for COPD©

Patients described the integral role of facilitators above and this was echoed in how the intervention should be delivered in the future. They described needing a facilitator to prompt reading and engagement with the manual and to help them make sense of it, with some suggesting more contact with the facilitator was required.

You’d need somebody else…what happens if I don’t understand something? (H15; hybrid sessions)

Additionally, patients felt that if the intervention was to be delivered digitally, facilitators would be needed to train and provide ongoing support to ensure its accessibility for patients.

Facilitators felt that their delivery could be improved by having a psychology practitioner within the team who could further support discussions of health behaviour change and psychological wellbeing management.

I think a psychologist would be great because it’s all about that change in behaviours for better health… because I did find that mental health and knocking down the walls of the behavioural change quite challenging. (HCP10; group sessions)

## Discussion

In this qualitative analysis of the SPACE for COPD© maintenance study, we explored the intervention’s acceptability and the impact of its implementation into the PR pathway. This was important because research shows us that implementing interventions into healthcare settings can be challenging, and therefore exploring and reacting to the context in which it situates is instrumental to its success.^17^ Analysis of interviews with study patients and focus groups with the healthcare professionals who delivered the intervention led us to generate six themes which explained the adoption and integration of positive COPD management strategies.

The findings show that goal setting activities and creating new exercise habits were key ingredients to the adoption and integration of positive COPD self-management. This compliments the backdrop of research into habit formation. Duhigg states that three ingredients need to be in place to create a “habit loop,” (1) frequent repetition, (2) recurring contexts and associated context cues, and (3) intermittent rewards.^18^ The intermittent rewards our patients described were the biopsychosocial outcomes which increased their motivation to set and achieve goals and to maintain their exercise habits. Similarly, a systematic review exploring the barriers and facilitators to physical activity post PR showed that as a result of people’s improved wellbeing their intentions, self-efficacy and perception of capabilities for physical activity also increased.^19^

Our findings spotlight one essential ingredient for the “habit loop”; context. Robinson and colleagues found that maintaining an environment (or a context) through opportunities to engage in activities and establish a routine were important to maintaining physical activity post PR.^19^ Whilst we were able to maintain some context for patients such as access to the manual and valued interaction with the facilitator, there were major changes to their context and associated context cues as a result of the COVID-19 pandemic. For example, our population was deemed clinically extremely vulnerable to COVID-19 and was therefore instructed to “shield” from the general population.^20^,^21^ Research conducted during this time reported that people living with COPD were significantly less active because they were no longer able to do activities out of the house (eg food shopping), attend face to face healthcare consultations^22^ or access exercise referral schemes and exercise spaces (eg gyms). As a result, people reported increased anxiety, stress, fear and uncertainty in how COVID-19 would impact their health and wellbeing.^23^ Likewise, there were changes to the facilitators’ context too. As a result of the COVID-19 pandemic some described feeling ill-equipped to deal with patients’ reactions to their changing context and wondered if they were appropriately skilled to discuss psychological wellbeing with patients. This is not the first time this concern has been raised^24^ and it therefore requires further consideration when updating our facilitator training.

Interestingly, some of our patients reported adaptations they made to continue to set and achieve goals and maintain their exercise habits. For some, this was swapping an outdoor or community-based activity to Joe Wicks “The Body Coach” programme as they could do this safely at home. According to Duhigg, the decision to adapt a new habit occurs when context changes and new behavior is deliberately considered.^18^ We found that patients who prioritised their health and wellbeing deliberately considered alternatives and were therefore able to make these adaptations. Likewise, we found that those who no longer prioritised their health and wellbeing because of the contextual change (ie COVID-19) did not deliberately consider alternatives or did not deem these alternatives acceptable to make adaptations. We propose that it is therefore essential for a PR maintenance intervention to develop and harness peoples’ motivation for managing health and wellbeing in changing contexts.

The personalisation of the intervention, as a result of the COVID-19 pandemic, meant a variety of intervention delivery models were adopted and patients were offered a choice of either one to one telephone sessions or online group sessions. Whilst this was not included in our original protocol and therefore the training provided to the facilitators, it was highly valued by patients as it enabled supported self-management during a particularly “scary time.” Furthermore, some patients, prompted by the patient-centred approach of their facilitator, were able to personalise their intervention further by adopting meaningful and realistic goals and exercise habits which fitted into their context. For example, by aligning their goal with a current hobby, by exercising with a family member, or by setting an overarching goal to spur them on to make smaller goals in order to reach this. This shows some patients’ considered their core values to guide their goal setting and exercise habit formation. This finding aligns with the self-activation hypothesis which posits that when a person’s values are used to guide their decision-making this is likely to result in meaningful behavior change.^25^ Therefore, our learning from this study is that our intervention needs to prompt and train people to re-evaluate their values when posed with a change in context.

Our findings highlight that future implementation of the intervention should embrace the personalisation we observed. Patients and facilitators proposed a menu of options (eg manual alone, telephone, group, telehealth, app-based) with differing levels of facilitator input and social support to enable people with differing needs and abilities to take up a maintenance intervention suitable for them. Patients and facilitators believed this would increase the delivery of holistic care and further embed the facilitator within a person’s healthcare team. This aligns with the National Health Service advancements in the digitisation of healthcare and healthcare services.^26^ However, it remains clear that not all patients find digital interventions accessible and/or acceptable. A menu of PR maintenance programmes should therefore include a combination of digital and non-digital interventions to account for the digital literacy of our population. To accommodate this menu, further training for facilitators is required to standardise the delivery via the differing methods. It is also worth exploring the impact of mode of delivery upon efficacy of the intervention.^27^ This need for personalisation has been mirrored in many interventions developed for people living with COPD. One example is the TANDEM study which measured a tailored psychological intervention for anxiety and depression management in people living with COPD. This was found to be ineffective, and it was postulated this was because it did not consider the wider and more complex aspects of peoples’ lives.^28^ Similarly, this need for personalisation is reflected in the PR literature. The BTS clinical statement reads that whilst supervised centre-based PR should be the first line treatment option, a menu of options is needed for those who decline or drop out of a centre-based programme.^5^

The strengths of this research are that we adopted a maximum variation approach to sampling which meant that we had a varied sample of patients to contribute to the interviews. This enabled us to capture perceptions from those who received differing doses of the intervention from the differing delivery methods. However, as some patients recalled, their perceptions and engagement with maintenance SPACE for COPD© maintenance was significantly influenced by the COVID-19 pandemic and should the study be conducted again many postulated that their adoption and integration of positive COPD management strategies may have been different. Our sampling method did not consider the severity of people's COPD or their symptom burden. These may have influenced our results. Furthermore, our nested study population was predominantly White British. Whilst this is reflective of our wider study’s patients, and the National Respiratory Audit Programme, it is not a reflection of our city’s population. Therefore, our findings are not widely generalisable.

## Conclusion

Our qualitative appraisal of maintenance SPACE for COPD© showed it was acceptable and could be implemented into a changing healthcare context during the COVID-19 pandemic. This implementation resulted in greater personalisation of the intervention and a menu of options. The intervention facilitators led focused discussions on behavioural activation and goal setting which supported people to adopt and integrate new exercise habits when they were able to adapt and personalise their activities to their home lives. Motivating and harnessing people's self-management skills within changing contexts are key to a PR maintenance intervention.
